# A bibliometric and visual analysis of epigenetic research publications for Alzheimer’s disease (2013–2023)

**DOI:** 10.3389/fnagi.2024.1332845

**Published:** 2024-01-16

**Authors:** YaPing Zhao, WenJing Ai, JingFeng Zheng, XianLiang Hu, LuShun Zhang

**Affiliations:** ^1^School of Clinical Medicine, Chengdu Medical College, Chengdu, China; ^2^Chengdu Eighth People’s Hospital, Geriatric Hospital of Chengdu Medical College, Chengdu, China; ^3^Sichuan Key Laboratory of Development and Regeneration, Department of Neurobiology, Chengdu Medical College, Chengdu, China; ^4^Department of Pathology and Pathophysiology, Chengdu Medical College, Chengdu, China

**Keywords:** Alzheimer’s disease, epigenetics, bibliometric analysis, visualization, neurology

## Abstract

**Background:**

Currently, the prevalence of Alzheimer’s disease (AD) is progressively rising, particularly in developed nations. There is an escalating focus on the onset and progression of AD. A mounting body of research indicates that epigenetics significantly contributes to AD and holds substantial promise as a novel therapeutic target for its treatment.

**Objective:**

The objective of this article is to present the AD areas of research interest, comprehend the contextual framework of the subject research, and investigate the prospective direction for future research development.

**Methods:**

ln Web of Science Core Collection (WOSCC), we searched documents by specific subject terms and their corresponding free words. VOSviewer, CiteSpace and Scimago Graphica were used to perform statistical analysis on measurement metrics such as the number of published papers, national cooperative networks, publishing countries, institutions, authors, co-cited journals, keywords, and visualize networks of related content elements.

**Results:**

We selected 1,530 articles from WOSCC from January 2013 to June 2023 about epigenetics of AD. Based on visual analysis, we could get that China and United States were the countries with the most research in this field. Bennett DA was the most contributed and prestigious scientist. The top 3 cited journals were Journal of Alzheimer’s Disease, Neurobiology of Aging and Molecular Neurobiology. According to the analysis of keywords and the frequency of citations, ncRNAs, transcription factor, genome, histone modification, blood DNA methylation, acetylation, biomarkers were hot research directions in AD today.

**Conclusion:**

According to bibliometric analysis, epigenetic research in AD was a promising research direction, and epigenetics had the potential to be used as AD biomarkers and therapeutic targets.

## 1 Introduction

Alzheimer’s disease (AD), a slowly progressive and irreversible neurodegenerative disease, is the most common form of dementia (60–70%). It mainly affects the elderly, causing cognitive impairment and some changes in mood and behavior. In March 2023, according to the AD-related data released by the WHO, there were currently more than 550,000 people with dementia in the world, and there were nearly 100,000 new cases every year. There is doubtless that AD has become a public health priority (World Health Organization, 2023).

Epigenetics refers to epigenetic modifications associated with the regulation of gene expression and differentiation, as well as genetic changes in gene activity or cellular phenotype, without altering DNA sequence (Hernández-Romero et al., 2019). Current studies had shown that epigenetic mechanisms including DNA methylation and hydroxy methylation, histone modifications, and regulation of non-coding RNAs (ncRNAs) were closely linked to AD and helpful for the treatment of AD (Wang et al., 2020; Wu and Kuo, 2020; Xiao et al., 2020; Lauretti et al., 2021; Nikolac Perkovic et al., 2021). At present, the research hotspots in epigenetics of AD are still DNA methylation, regulation of miRNAs, histone acetylation, etc. Studies have shown that many microRNAs (miRNAs) can be used as markers for early diagnosis of AD, and epigenetic mechanisms regulate gene expression in preliminary AD (Gao et al., 2022). Therefore, studying the interaction between AD and epigenetics plays an important role in the treatment and prevention of AD. However, the pathogenesis of AD is complex, and existing drugs have extreme side effects, so there has been no considerable progress in the treatment of AD. Therefore, it is still very important to further elucidate the epigenetic mechanism of AD, find newer and more reliable biomarkers, propose new treatment strategies, and develop fresh research directions. Meanwhile, there is no bibliometric analysis in this field. Therefore, in order to facilitate researchers to better understand the current state and development trend of the link of epigenetics and AD, we used bibliometric methods to quantitatively analyze and visualize the literature in this field.

## 2 Methods

### 2.1 Data sources and search strategies

**Figure 1:**
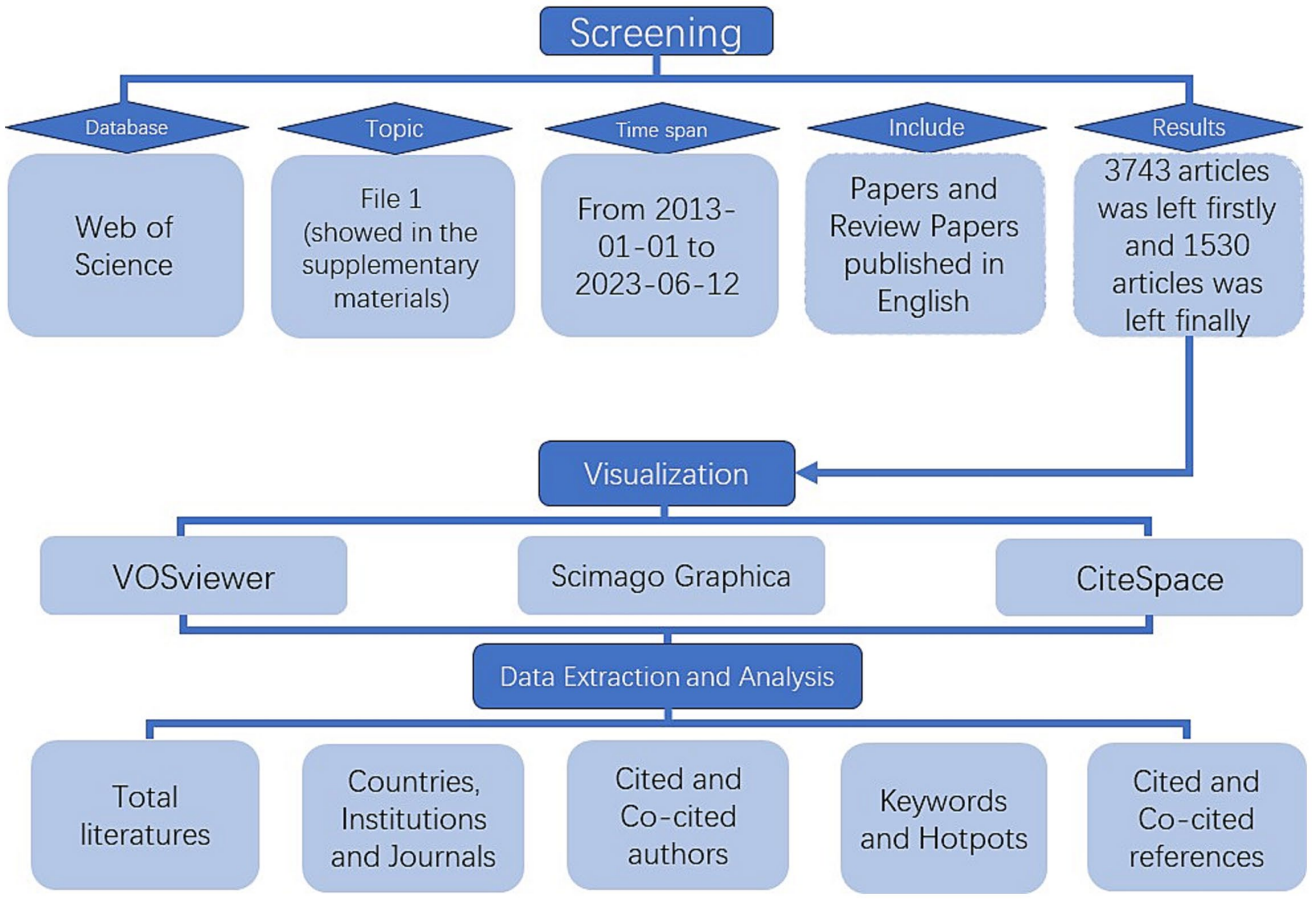
Article retrieval and process flow chart.

All articles from January 1, 2013 to June 12, 2023 were searched in WOSCC using File 1 (showed in the Supplementary materials) as the screening strategies. We restricted the language type of the article to English, and included papers and review papers. 3,743 articles were obtained. Then we excluded articles that did not fit the topic by reading the article title, abstract and even the full text of the paper, and finally we retained 1,530 papers. The specific retrieval strategy for this study could be found in Figure 1.

### 2.2 Analysis tools

Data is imported into VOSviewer.1.6.18 and CiteSpace.6.2.3 to visualize the co-citation network maps of countries, institutions, authors, journals, keywords and articles and analyze relevant publication and citation data.

Bibliometrics is a discipline concerned with the study of distributional structure, quantitative relationship, change rule, and quantitative management of bibliometrics and data, and goes on to discuss some of the structures, characteristics and laws of science and technology using quantitative research methods such as numbers and statistics.

VOSviewer, developed by Leiden University in the Netherlands, is good at generating any type of text map, and can perform publication statistics, cooperative network analysis, co-occurrence analysis, and co-citation analysis on documents (Van Eck and Waltman, 2010).

CiteSpace is a visual bibliometric analysis software developed by Professor Chaomei Chen, which can be used for cooperative network analysis and clustering. In addition, the centrality and explosive display is its most prominent advantage (Synnestvedt et al., 2005). Centrality refers to the ability of an entity to connect the content of different groups together, revealing its role as a turning point in the entire network. The higher the centrality of a node, the more times it appears on the shortest path in the overall network, and the greater its influence and importance. “Citation bursts” refers to the sudden increase in the frequency of a specific type of event or the frequency of a large number of citations during a specific time period. Through centrality and bursting analysis, we can quickly understand the knowledge structure, research hotspots and frontiers, and the evolution of emerging topics. “Strength” indicated the intensity of the burst, “Begin” indicated the start year of the burst, and “End” indicated the end year of the burst. The red bar indicated the duration of the burst, and the blue bar indicated the entire period from 2013 to 2023 (Chen et al., 2014).

## 3 Results

### 3.1 Analysis of publication output

Through our search, we acquired 1,530 English-language articles in the field of epigenetics of AD including papers and review papers. As shown in Supplementary Figure 1, the number of publications experienced a steady increase from 66 in 2013 to 229 in 2022. 2013 to 2018 was the first stage of primary development, during which number of articles in this field grew with stability and was correspondingly few, demonstrating progress in this period had been slow and there was no further breakthrough. In 2019–2022, the publications on epigenetics of AD research went through a second phase of high-speed development. Despite a slight decline in publications in 2018 and 2021, the cumulative number of publications over the decade reached 1,442, averaging roughly 144 per year.

### 3.2 Analysis of country

**Figure 2:**
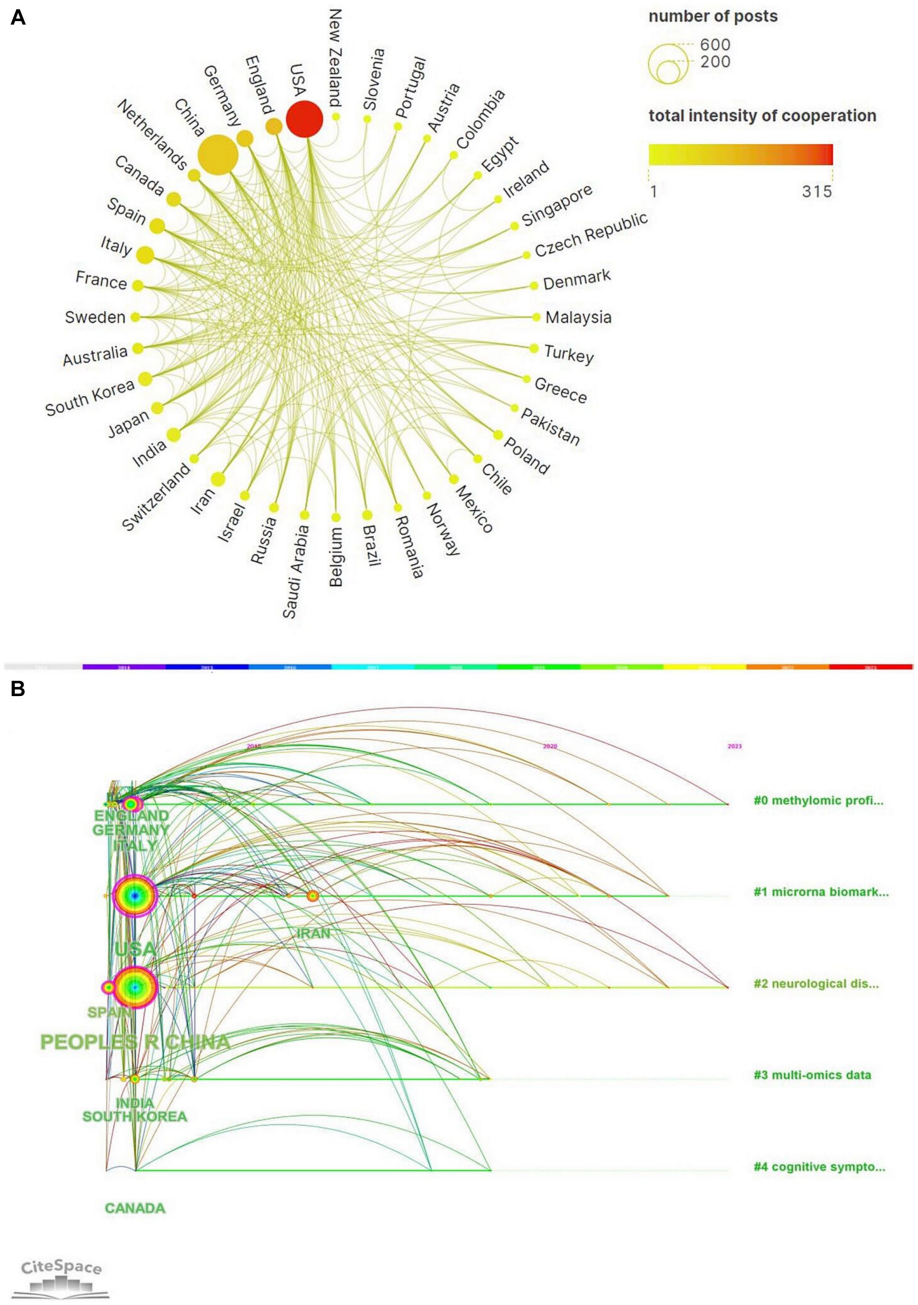
**(A)** A world map of the intensity of cooperation among 20 countries. **(B)** CiteSpace-based visualization of national timeline diagram.

From January 1, 2013 to June 12, 2023, a total of 76 countries had participated in epigenetics of AD. The top 10 countries’ documents, citations, average citation/publication and centrality were showed in the Table 1. It showed that People’s Republic of China ranked first with 559 publications and followed by USA with 474 publications. USA had the most citations (20,321 citations) and the most average citations (Berson et al., 2018; Table 1). This can be inferred that China and USA were the countries with the most research related to epigenetics of AD and China, USA, Italy, Germany, Spain with centrality more than 0.1 suggested that they had strong academic influence in this field. Figure 2A showed collaborative networks between countries. A node in the figure represented a country and the size of node represented the corresponding country publication number and the lines between nodes represented collaborative relationships, which indicated China and USA were most closely linked in this regard. The colors represent the total intensity of cooperation between countries in this field. Figure 2B showed the national timeline diagram based on CiteSpace. The colored circles on one of the lines formed a cluster, and a total of five clusters were formed finally. A circle represented a country, the size of the circle represented the publication volume of the country, and the lines between the circles represented the partnership. The order of the circles represented the time when the country began research on epigenetics of AD. The purple ring in the circle represented the centrality of the country, and the thickness of the purple ring described the magnitude of the centrality. According to this, we could find that USA, Chinese and Spain had the greater centrality. England, Germany, Italy, USA, Spain, People’s Republic of China, India, South Korea, Canada started research earlier and Iran started later. It also reflected that the research on neurological disease and methylomics had lasted for a long time and they were hot topics.

**Table 1:** Top 10 countries’ documents, citations, average citation/publication and centrality.

Rank	Country	Documents	Citations	Average citation/Publication	Centrality
1	People’s Republic of China	559	13,777	25	0.13
2	USA	474	20,321	43	0.80
3	Italy	99	3,964	40	0.10
4	England	87	2,952	34	0.09
5	Germany	87	3,968	46	0.11
6	Spain	71	2,027	29	0.14
7	Canada	60	1,914	32	0.05
8	Iran	60	885	15	0.06
9	South Korea	56	1,623	29	0.00
10	India	56	839	15	0.05

### 3.3 Analysis of institutions

**Figure 3:**
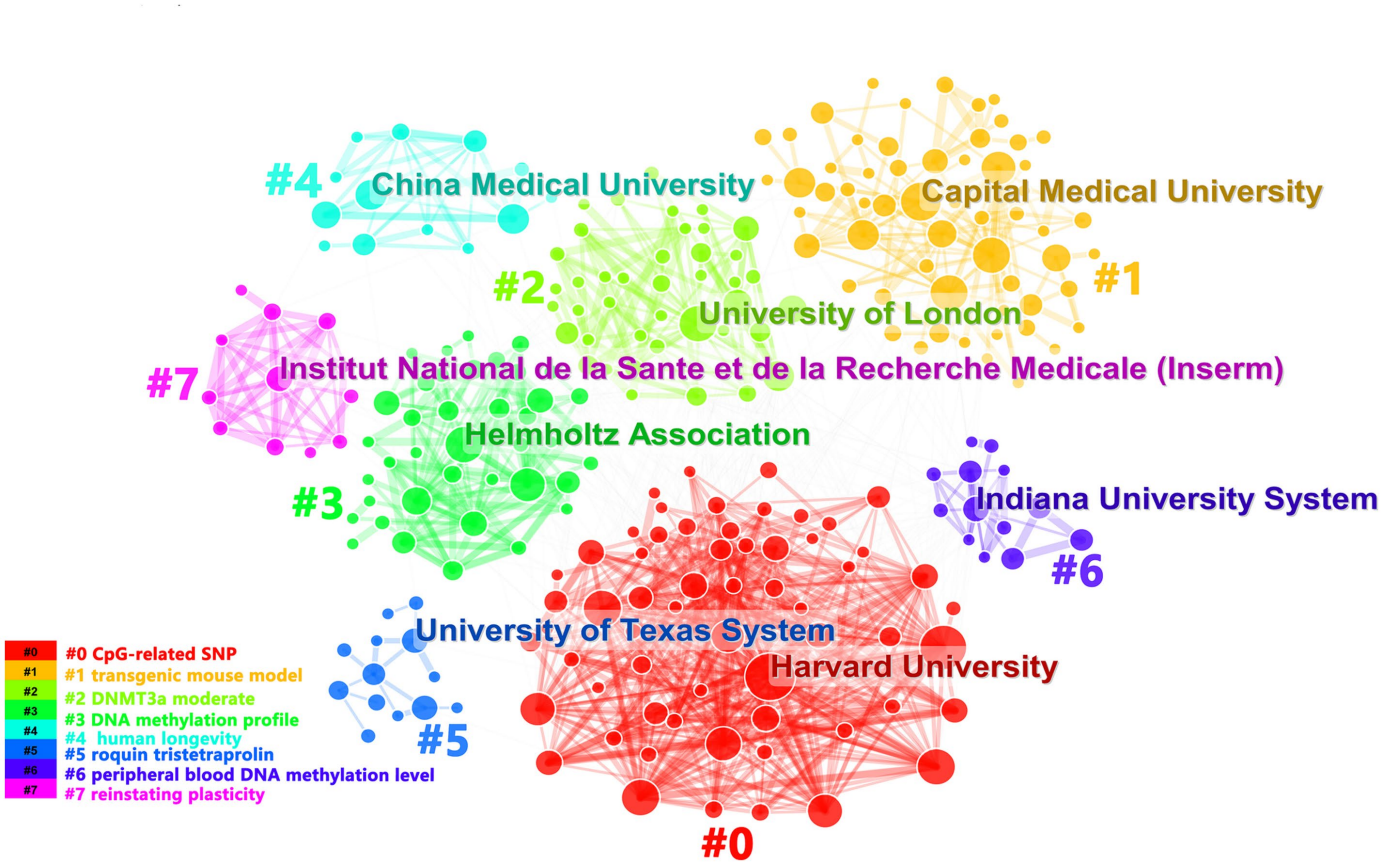
It showed organization cooperation clusters. Each cluster described the main institution and the topics of the cluster were shown in the corresponding legend.

We had analyzed productions of coming from 1899 institutions. We found that Capital Medical University’s documents accounted for the most. But Maastricht University had the most citations (Supplementary Figure 2A). It showed that Maastricht University’s documents were of high quality. Representing the 4 nodes of Harvard University, Shanghai Jiao Tong University, Rush University, Columbus University had been presented dense lines, which indicated that they cooperated with other institutions frequently (Supplementary Figure 2B). As shown in Figure 3, we could also find that among the clusters, the clusters represented by Harvard University had denser networks. Maybe the direction studied by Harvard University was recognized by most of institutions. The institutions were divided into 8 clusters according to title words and the colors of the clusters corresponded to the numbers #0–7 in the color bar in Figure 3.

### 3.4 Analysis of authors and authors’ co-citations

**Figure 4:**
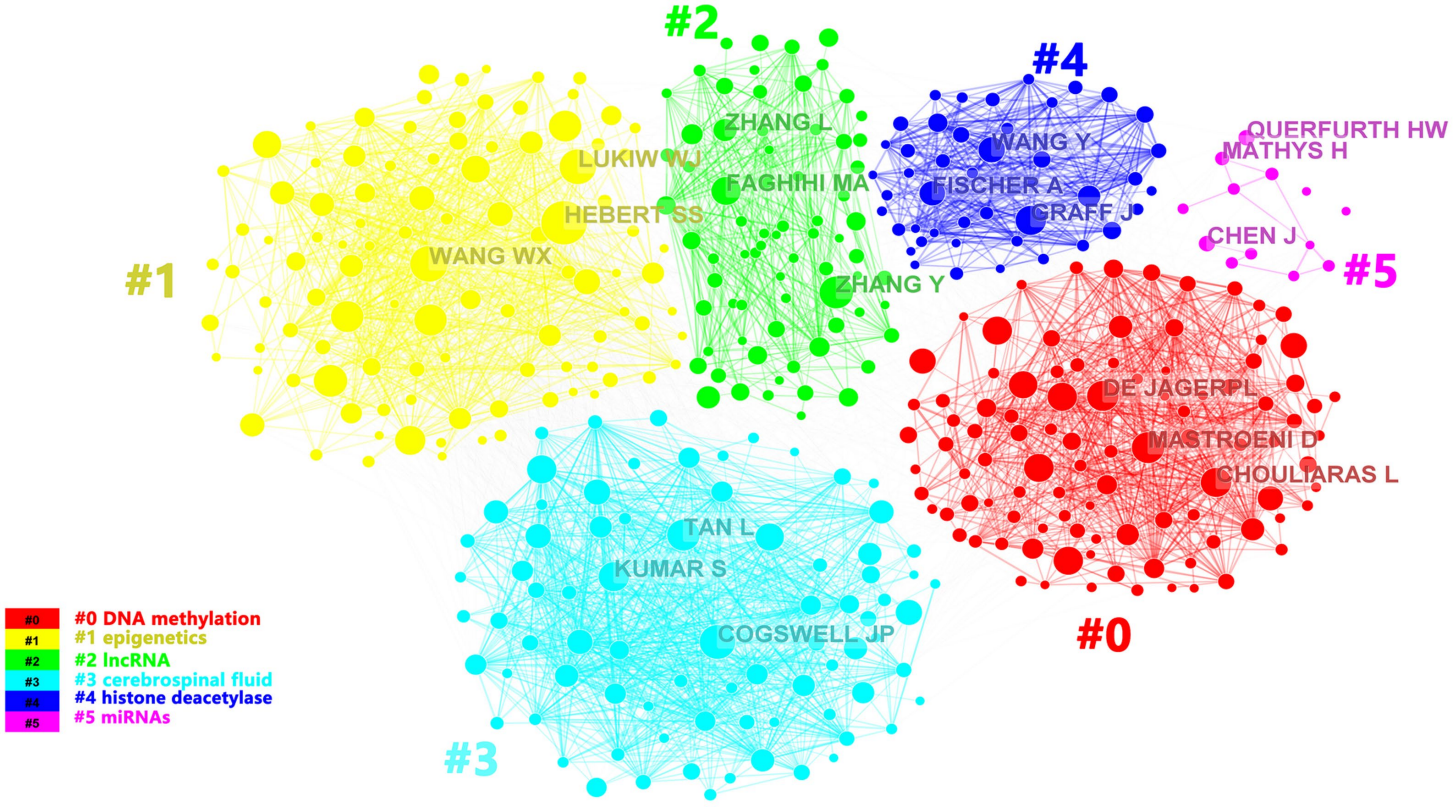
The cluster visualization map of topics for the authors. The colors of the clusters corresponded to the numbers #0–5 in the color bar and the topics of the cluster were shown in the corresponding legend.

A total of 44,213 authors had devoted to the research of epigenetics of AD over the past 10 years. We listed the top 11 most productive authors according to the documents, citations and average citation/publication (Table 2). The top 5 productive authors were Bennett DA, De Jager PL, Lukiw WJ, Lunnon K and Zhang W. The top 5 cited authors were Bennet DA, Lukiw WJ, Zhang W, Mill J and Zhao YH. As another parameter, average citation/publication was used to assess authors’ contribution, which was able to better reflect the quality of the author’s publications. Due to the highest number of posts and highest citation count, it had no doubt that Bennett DA was the most contributed and prestigious scientist in this field. Partnerships between authors were evaluated by collaboration network visualization of co-authors. As shown in Figure 4, the authors were divided into 6 clusters according to keyword relevance and cooperation strength. The color of the clusters corresponded to the numbers #0–5 in the color bar in Figure 4. Every node was regarded as a symbol of different author while the circle size reflected the number of articles published by each author. The thicker lines between the circles indicated the closer connection linking the writers.

**Table 2:** Top 11 cited authors’ documents, citations and average citation/publication.

Rank	Author	Documents	Citations	Average citation/publication
1	Bennett DA	26	1,360	52
2	De Jager PL	21	589	28
3	Lukiw WJ	19	836	44
4	Lunnon K	17	602	35
5	Zhang W	16	698	44
6	Mill J	14	650	46
7	Zhao YH	12	629	52
8	Kumar S	12	603	50
9	Reddy PH	12	570	48
10	Liu R	12	157	13
11	Pallas M	12	413	34

### 3.5 Analysis of journals

All articles were distributed in 5,437 journals and Table 3 displayed the top 10 journals ranked by citation, documents and average citation/publication. In terms of the number of citations, the top 3 were Journal of Alzheimer’s Disease, Neurobiology of Aging and Molecular Neurobiology; In terms of the number of documents, the top 3 were Journal of Alzheimer’s Disease, International Journal of Molecular and Molecular Neurobiology; The impact factors (IF) of various journals in 2022 were also shown in Table 3, with the most influential journals being Aging-US, Cells. With no doubt that Journal of Alzheimer’s Disease was the most productive journal, but Aging-US was the most influential journal with the highest average citation/publication and IF.

**Table 3:** The top 10 journals ranked by citations, documents, and average citation/publication and IF.

Rank	Journal	Citations	Documents	Average citation/publication	IF
1	Journal of Alzheimer’s Disease	2,804	85	33	4.0
2	Neurobiology of Aging	1,744	30	58	4.2
3	Molecular Neurobiology	1,732	49	35	3.1
4	Aging-US	1,693	21	81	6.0
5	International Journal of Molecular	1,223	58	21	5.6
6	Scientific Reports	836	30	28	4.6
7	Frontiers in Aging Neuroscience	784	47	17	4.8
8	Frontiers in Neuroscience	761	26	29	5.3
9	Frontiers in Molecular Neuroscience	573	22	26	4.8
10	Cells	166	22	8	6.0

### 3.6 Analysis of keywords

**Figure 5:**
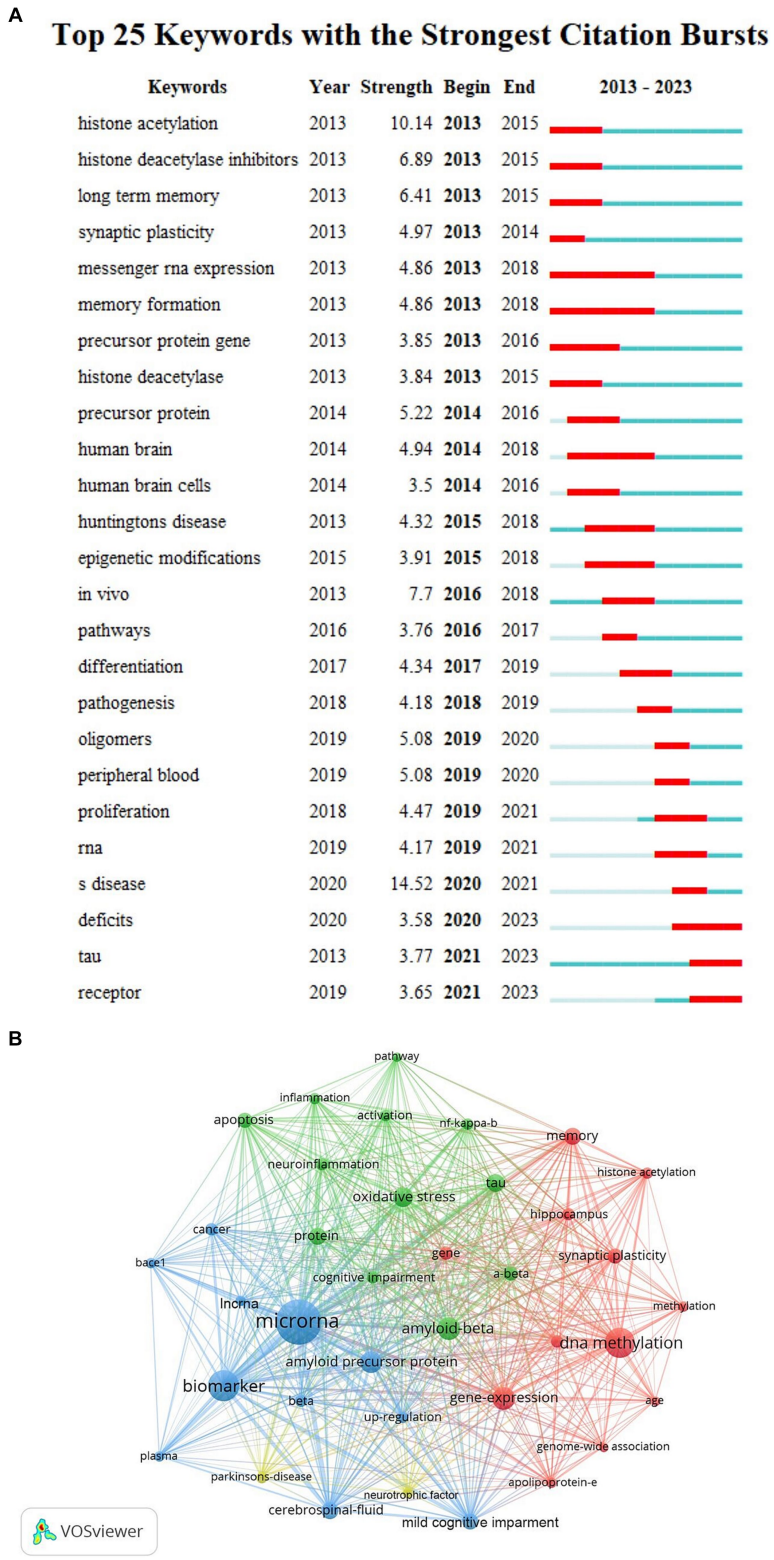
**(A)** Top 20 keywords with the strongest citation bursts. The strongest citation bursts time period was indicated in red. **(B)** The co-occurrence analysis of keywords based on VOSviewer. The size of the circle indicated the frequency of the keyword, different colors represented different clusters, the lines between nodes showed the correlation between keywords, the more lines the keywords distribute, and the relationship between this keyword and other keywords was stronger.

**Figure 6:**
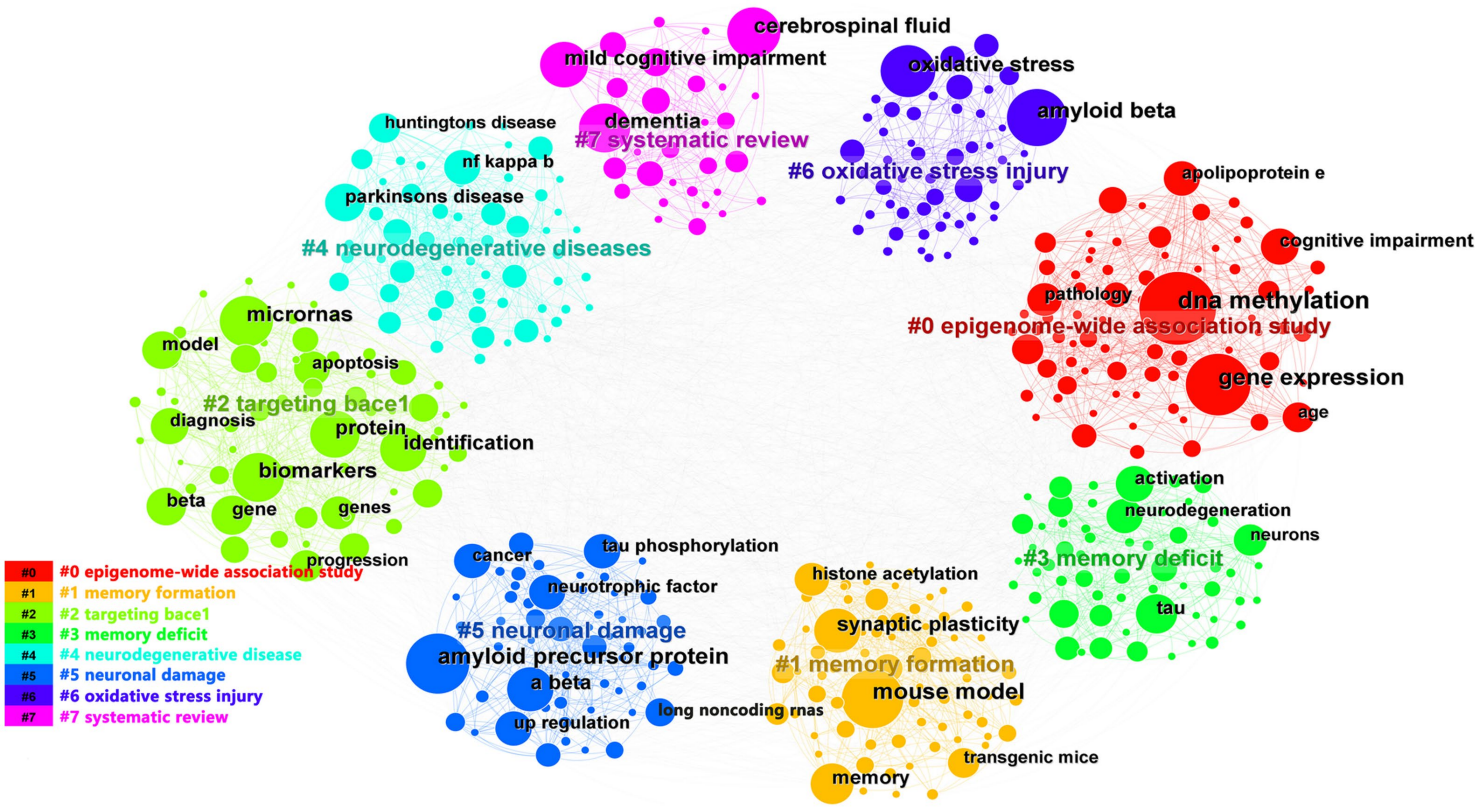
The co-occurrence analysis of keywords based on CiteSpace. The keywords were grouped into 8 clusters by topic and labeled with different colors. The topics of the cluster were shown in the corresponding legend.

We used CiteSpace and VOSviewer to visualize the 5,533 keywords of 1,530 selected articles. The top 10 frequently occurring keywords were miRNAs (486), biomarkers (273), DNA methylation (257), amyloid beta (168), gene expression (165), amyloid precursor protein (160), oxidative stress (134), neurodegeneration (123), tau (116), cerebrospinal-fluid (110). And DNA methylation’s centrality (0.05) was the most, which indicated it had the greatest influence among them (Table 4). Based on the strength of the citation bursts, we got the top 25 keywords. CiteSpace conducted a burst detection of keywords. “Strength” indicated the intensity of the burst, “Begin” indicated the start year of the burst, and “End” indicated the end year of the burst. The red bar reflected the duration of the burst, and the blue bar reflected the entire period from 2013 to 2023. The strength of histone acetylation and histone deacetylase inhibitors’ (HDACI) citation bursts had been the highest in the past years. The figure also showed the current higher strength of deficits, tau, and receptor, indicating that they have been the focus of research in recent years (Figure 5A). According to the network visualization showed in the Figure 5B, the size of the circle indicated the frequency of the keyword, different colors represented different clusters, the lines between nodes showed the correlation between keywords, the more lines the keywords distribute, and the relationship between this keyword and other keywords was stronger, we found that apart from Alzheimer’s and epigenetics themselves, keywords related to miRNAs, biomarkers, DNA methylation appeared more. As shown in Figure 6, the keywords were grouped into 8 clusters by topic and labeled with different colors. The first group was epigenome-wide association studies (cluster #0). The keywords were DNA methylation, gene expression, cognitive impairment, apolipoprotein E and pathology. This group mainly studied the role of epigenetic gene modification in the pathogenesis of AD. The second group was memory formation (cluster #1) and involved keywords like transgenic mouse models, histone acetylation, synaptic plasticity and memory. This group mostly explored the effects and mechanisms of AD on memory. The third group was targeting β-secretase (BACE1) (cluster #2) and this group contained miRNAs, biomarkers, apoptosis and genes. It showed that epigenetic mechanisms apply to targeted therapy of AD. The fourth group was memory deficit (cluster #3), which also involved keywords such as tau, neurodegeneration, indicating that abnormal post-translational modifications of tau protein was related to memory deficit in AD. The fifth group was neurodegenerative disease (cluster #4) and the keywords were Parkinson’s disease and Huntington’s disease and illustrated the association between AD and other neurodegenerative diseases in terms of epigenetics. The sixth group was neuronal damage (cluster #5) and involved keywords like amyloid precursor protein, tau phosphorylation, neurotrophic factor and lncRNAs, which revealed the mechanism of neuronal damage. The seventh group’s keywords were mainly oxidative stress and amyloid beta (cluster #6). This group of studies revealed oxidative stress and neuroinflammation activation pathways in AD. The eighth group was systematic review (cluster #7).

**Table 4:** Centrality of the top 10 keywords in frequency.

Rank	Keywords	Centrality	Frequency
1	miRNAs	0.03	441
2	biomarkers	0.02	273
3	DNA methylation	0.05	257
4	amyloid beta	0.03	168
5	gene expression	0.02	165
6	amyloid precursor protein	0.02	160
7	oxidative stress	0.01	134
8	neurodegeneration	0.03	123
9	tau	0.02	116
10	cerebrospinal-fluid	0.03	110

### 3.7 Analysis of cited and co-cited references

**Figure 7:**
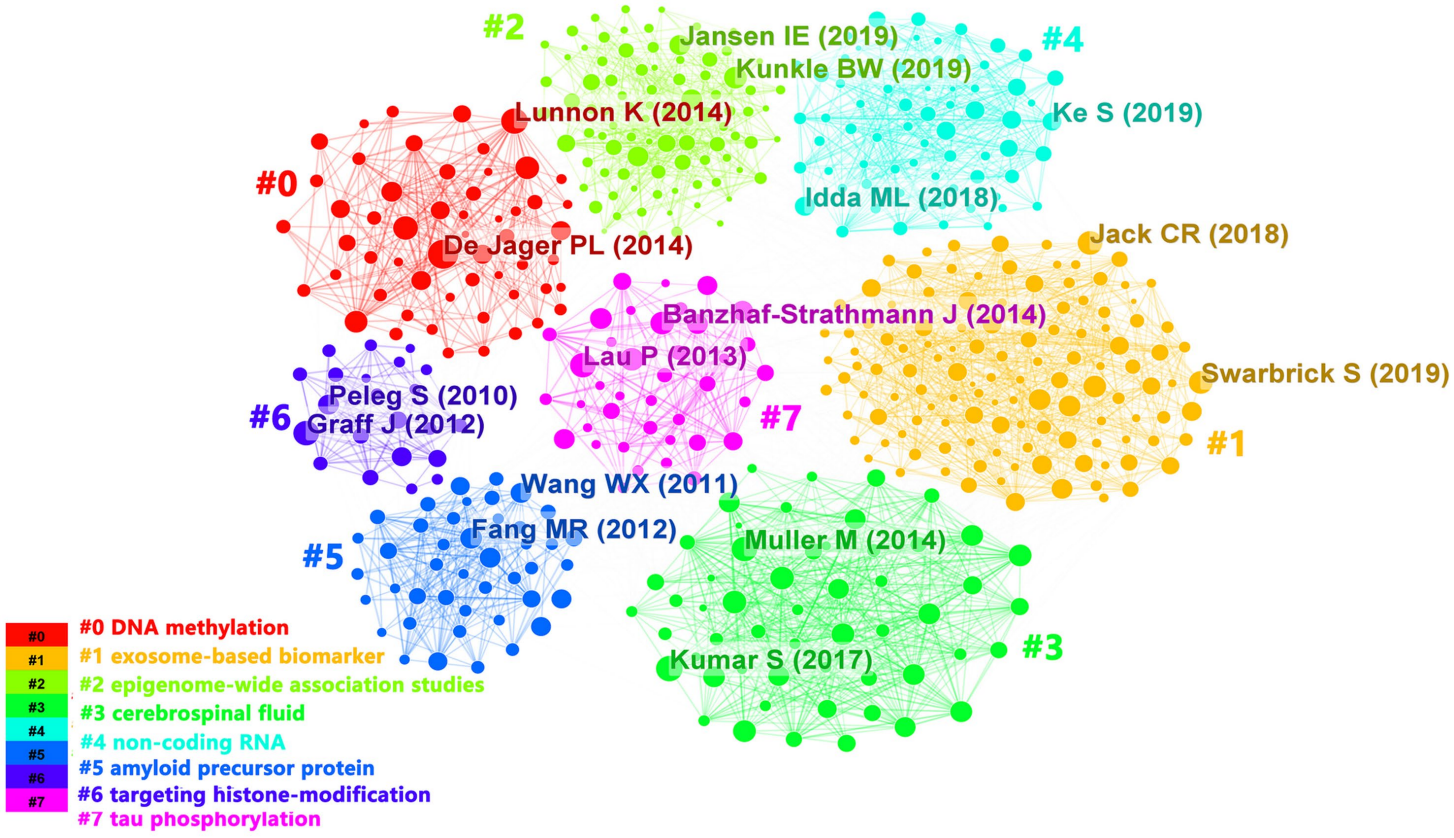
The clustering visualization map of topics for cited references. The colors of the clusters corresponded to the numbers #0–7 in the color bar and the topics of the cluster were shown in the corresponding legend.

In the past 10 years, a total of 1,530 articles have been cited in epigenetics of AD research field, with 67,138 citations and an average of 44 citations per document. As shown in Figure 7, references were divided into 8 clusters according to topics and marked with diverse colors. The color of the clusters corresponded to the numbers #0–7 in the color bar in Figure 7. #0, #1, and #2 had the largest clusters, indicating that the 3 areas of DNA methylation, exosome-based biomarker, and epigenome-wide association studies have been cited and studied the most in recent years.

## 4 Discussion

Based on the co-occurrence analysis and keywords burst graph analysis of CiteSpace and VOSviewer, we found that keywords describing ncRNAs, transcription factor, genome, histone modification, blood DNA methylation, acetylation, biomarkers have been used most frequently in recent years. In addition, we should also pay attention to macroscopic mechanisms such as oxidative stress, synaptic plasticity, and apoptosis.

In terms of some correlative studies, several highly cited articles highlighted the main research directions in epigenetics of AD, which engrossed our attention. For example, Kumar et al. (2013) initially generated a compilation of prominent canonical pathways through the utilization of the Ingenuity Pathway Analysis platform. They discerned essential circulating miRNAs pathways that exhibited biological associations with the biology of AD. These pathways encompassed axonal guidance signaling, ephrin receptor signaling, actin cytoskeleton signaling, clathrin-mediated endocytosis signaling and rhoA signaling (Gallo, 2007; Lin et al., 2009; Wu and Yao, 2009; Chacon et al., 2011). Additionally, they demonstrated that a canonical pathway associated with TypeII Diabetes Mellitus signaling was notably enriched with targets of the signature miRNAs. “An epigenetic biomarker of aging for lifespan and healthspan (2018)” written by Levine mentioned they developed a new epigenetic biomarker of aging-DNAm Phenoage, using an innovative two-step process (Levine et al., 2018). The study conducted by Gjoneska et al. examined various aging outcomes, including all-cause mortality, cancer, health span, physical function, and AD. The researchers found that their evaluation method had notable advantages over previous measurements in predicting these outcomes. Specifically, they investigated the transcriptional and chromatin state dynamics in early and late hippocampal pathology in a mouse model of AD-like neurodegeneration (Gjoneska et al., 2015). Their study revealed a synergistic downregulation of genes and regulatory regions associated with synaptic plasticity, while immune response genes and regulatory regions were upregulated, suggesting that immune processes also play an important role in the progression of AD. When cells in the hippocampus, a region closely associated with AD, lose function or die, new connections can be made through healthy neurons within the population, and these connections can either exhibit parallel function (homologous sprouting) or fibers from the convergent pathway (heterologous sprouting) can maintain functional stability through enhanced or weakened signals, this result demonstrates the plasticity of synapses in the hippocampus (Cotman and Anderson, 1988). Epigenetic markers that are closely related to the development of AD, such as DNA and histone modifications, have been found in the peripheral blood of AD patients in clinical studies, and considering the reversibility of epigenetic modifications and the complexity of gene–environment interactions, we may need to combine epigenetic therapeutics with environmental strategies and drugs with multiple targets (Jeremic et al., 2023). The above studies indicate that based on the involvement of various epigenetic mechanisms in the development and pathogenesis of AD, the study of new AD biomarkers and targeted therapies is a major research direction in this field. To further advance, it is crucial to enhance our understanding of the fundamental biology of aging, particularly in terms of elucidating the interplay between cell type specificity and aging biology. Additionally, efforts such as the Model Biological Development and Evaluation of Late-Onset Alzheimer’s Disease should be undertaken to refine the model system (Oblak et al., 2020; Gonzales et al., 2022).

**Figure 8:**
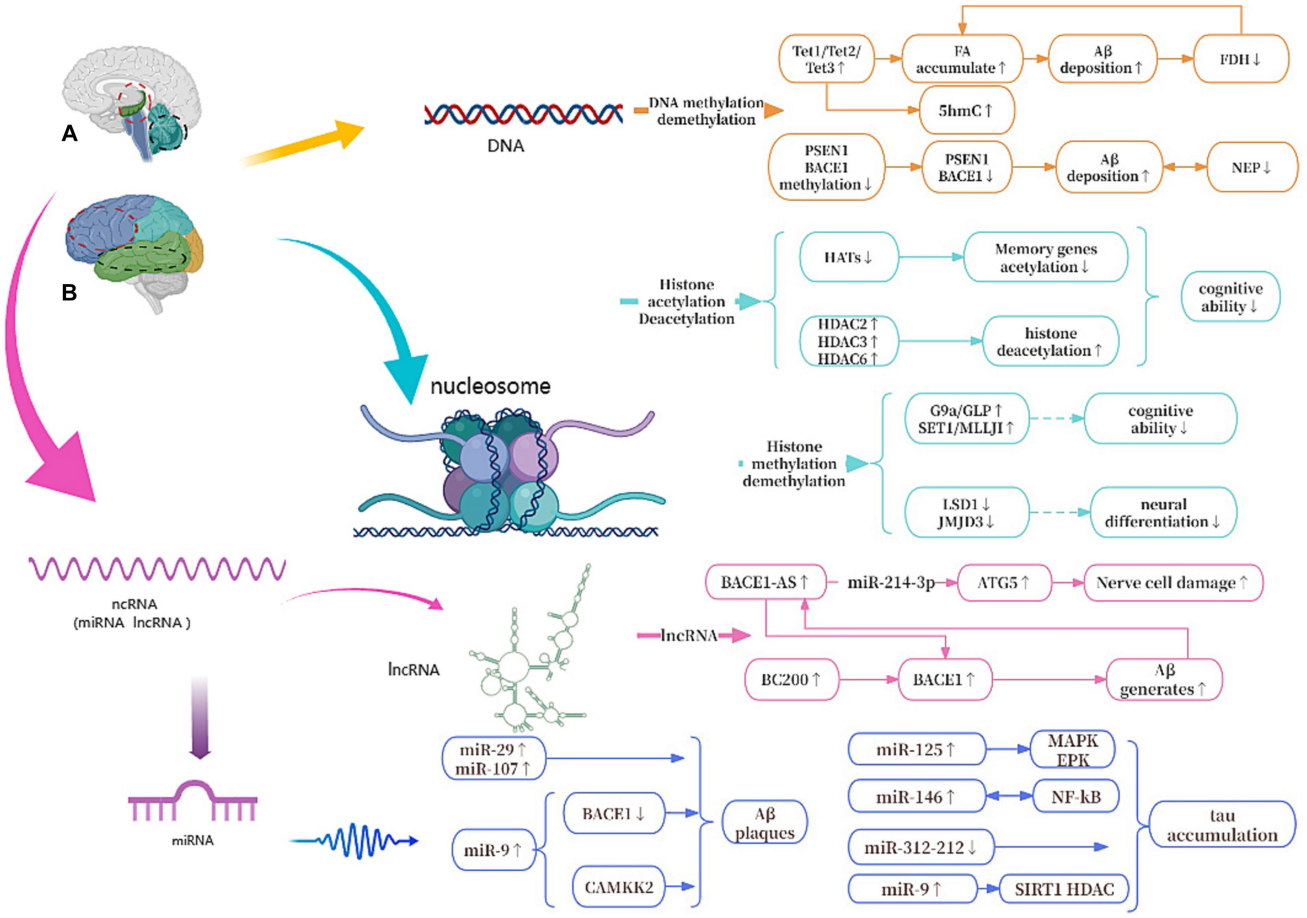
Epigenetic mechanisms of AD. **(A)** The red circle represents the hippocampus, and the black circle represents the cerebellum. **(B)** The red circle represents the frontal lobe, and the black circle represents the temporal lobe. Tet1, Tet2, and Tet3, All Tet proteins regulate DNA demethylation in the body; FA, formaldehyde; FDH, formaldehyde dehydrogenase; PSEN1, presenilin 1; BACE1, beta-site APP cleaving enzyme 1; NEP, neprilysin; G9a/GLP, Both are histone methyltransferase; LSD1/JMJD3, Both are histone demethylase; HATs, histone acetylases; HDACs, histone deacetylases; ATG5, autophagy-related genes 5; BC200, brain cytoplasmic 200; MAPK/ERK, protein kinase/extracellular signal-reduced kinases; NF-κB, nuclear factor κB; CAMKK2, calcium/calmodulin-dependent protein kinase 2.

As shown in Figure 8, we will systematically elucidate the epigenetic mechanisms of AD from three aspects of DNA, histone modifications and ncRNAs.

### 4.1 DNA methylation and demethylation

Among all the known mechanisms of epigenetics, DNA methylation has been studied and known the most. During DNA methylation, a methyl group is added to the 5th atom of the cytosine ring, which forms 5-methylcytosine (5mC) (Prokhortchouk and Defossez, 2008). Oliveira et al. found that DNA methyltransferases3a2 (Dnmt3a2) reduced in the hippocampus of mice, and the restoration of Dnmt3a2 recovered the cognitive functions (Moore et al., 2013; Lee et al., 2014). This shows to some extent that methylation of DNA affects gene expression and may hinder the binding of transcription activators to DNA (Lee et al., 2014), its precise regulation is essential for maintaining normal cognitive function (Moore et al., 2013). There are three Tet proteins that regulate DNA demethylation in mammalian bodies and modify DNA by repeatedly oxidizing 5mC to produce 5-hydroxymethylcytosine (5hmC): Tet1, Tet2 and Tet3, which regulate the expression of different genes in different cells to control cellular behavior (Zhang et al., 2023). Tet1 is currently thought to play the most important role in AD. Disruption of Tet1 and subsequent disruption of the methylome can alter AD-associated hydroxy methylation, as well as the expression of key genes involved in maintaining the epigenome, neuronal health, and the activation and function of microglia (Armstrong et al., 2023). In addition, during DNA demethylation, the active substance formaldehyde (FA) is produced, and excessive exposure to FA or endogenous FA metabolism disorder can lead to abnormal accumulation of FA (Li et al., 2021). Excessive FA interacts with Aβ42 to accelerate Aβ aggregation, which in turn inhibits the activity of formaldehyde dehydrogenase (FDH), thereby reducing FA degradation and leading to FA accumulation (Fei et al., 2021). At the same time, Aβ plaque deposition can cause hypermethylation of Aβ-degrading enzymes such as neutral endopeptidase (NEP) and down-regulate the expression of NEP, while low expression of NEP will promote further deposition of Aβ (Lin et al., 2021). In addition, some studies have confirmed that S-adenosylmethionine via improving Presenilin-1 (PSEN1) and BACE1 expression reduces tau phosphorylation and Aβ in a mouse model (Cotman and Anderson, 1988; Zhang et al., 2023).

Relatively speaking, the study of DNA methylation is more mature, involving different areas of the blood, cerebrospinal fluid and brain. However, currently there is no evidence that DNA methylation in peripheral blood cells is associated with cerebral cortex samples (Yu et al., 2016), so the connection between blood and DNA methylation in the brain needs other clues. DNA methylation usually occurs in CpG islands within these gene promoter regions, which are long repeating sequences containing cytosine-guanine nucleotides (Xie et al., 2023). Studies have found that 5mC at certain CpG check points is highly correlated with age; therefore, biological age can be accurately predicted using relatively few CpG check points. Based on this, this modification can be used as an estimated indicator of an organism’s aging, called the epigenetic clock (Gao et al., 2022; Jeremic et al., 2023), which is also widely concerned.

Of course, there are also a small number of new studies that suggest that AD is associated with a decrease in bioenergy caused by mitochondrial dysfunction (Wallace, 2011; Coskun et al., 2012; Hroudová et al., 2014; Swerdlow et al., 2014), such as in one study, patients with low Aβ and high t-tau AD biomarkers and pre-symptomatic patients with PSEN1 mutation showed low levels of mitochondria in cerebrospinal fluid (Podlesniy et al., 2020). Mitochondrial DNA methylation is also regulated by epigenetic mechanisms, whether it is related to AD is also receiving increasing attention (Huang et al., 2022). However, research in the emerging field of mitochondrial DNA methylation is quite limited. For example, there are few specific displays of this epigenetic inheritance in the study of mitochondrial DNA. It is difficult to find the concentrated presentation area of mitochondrial DNA methylation, or to use means to present it, which will be a difficult problem (Bellizzi et al., 2013; Blanch et al., 2016).

### 4.2 Histone modifications

Histone modification mainly refers to the modification that occurs at the amino tail end of histone translation, such as methylation, acetylation, phosphorylation, ubiquitination, etc., which not only occurs in nerve cell development, but also plays an important role in the aging brain, and in the pathogenesis of AD (Mastroeni et al., 2011; Berson et al., 2018; Geng et al., 2021). Among them, histone acetylation is the most studied, that is, under the catalysis of histone acetyltransferase (HAT), the acetyl group in cofactor acetyl-CoA is added to the amino acid residues (mainly lysine in H3 and H4) at the N-terminal tail of histone, and then transcription begins (Santana et al., 2023). During this process, changes in related enzymes reduce histone acetylation levels, lead to inactivation of memory-related genes, and cause abnormal phosphorylation of tau, which in turn leads to cognitive degeneration (Peixoto and Abel, 2013).

The inhibition of histone deacetylases (HDACs) can reverse these changes and alleviate cognitive impairment (Gao et al., 2022). For instance, sodium phenylbutyrate, an HDACI, can induce the expression of neurotrophic factors through the protein kinase C-cAMP-response element binding protein pathway, thereby alleviating memory impairment in transgenic AD mice (Yang et al., 2017). Previous research has shown that proteomic analysis revealed a higher number of changes in histone post-translational modifications in the brains of AD patients compared to healthy elderly brains. Specifically, a significant increase in Histone H3 lysine is acetylated at site 27 (H3K27ac) and Histone H3 lysine is acetylated at site 9 (H3K9ac) was observed (Creyghton et al., 2010; Lu et al., 2014). Disease-specific alterations enriched for single-nucleotide polymorphisms from AD Genome-Wide Association Studies (GWAS) and expression quantitative trait loci points to potential involvement of H3K27ac in AD, indicating a possible disease-specific increase (Lambert et al., 2013; Kunkle et al., 2019; Xiong et al., 2023). Integrated multi-omics data reveals that H3K27ac and H3K9ac may play a role as potential epigenetic drivers in AD (Nativio et al., 2020). At the same time, recent epigenome studies have revealed the potential relationship between epigenome information loss and apoptosis of neurons and other cells. Although GWAS has revealed more than 40 genetic loci associated with AD, because more than 90% of risk loci are located in non-coding regions, transcriptome data alone is difficult to pinpoint the disease-causing loci of AD and elucidate its molecular mechanisms (Bellenguez et al., 2022). For example, using this approach, the researchers revealed the global epigenomic information loss that occurs with H3K27ac histone modifications in patients with advanced AD; Runt-associated transcription factor 1, Spi-1 Proto-Oncogene and other transcription factors play an important role in the pathogenesis of microglia AD (Xiong et al., 2023). It can be seen that studies at the multi-omics level play an important role in revealing the combined effects at the cellular and molecular levels in AD. Therefore, integrating epigenome and transcriptome data may be the key to solving the difficulties of neurodegenerative diseases such as AD.

Histone methylation refers to the process of adding methyl groups to the lysine or arginine residues of histones, this process is catalyzed by histone methyltransferases (HMT) that catalyze the transfer of 1–3 methyl groups to histones (Zhou and Shao, 2021; Ding et al., 2023). The effect of histone methylation on transcription is determined by several factors, including the specific histone involved, the specific residues that are modified, and the overall degree of methylation at each site. Typically, methylation of H3K4, H3K36, and H3K79 is associated with active transcription, while methylation of H3K9, H3K27, and H4K20 is associated with inhibitory transcription (Yi and Kim, 2020).

G9a is a type of histone methyltransferase with a SET domain. They are involved in the *in vivo* modification of H3K9me2. H3K9me2 is the fourth lysine of histone H3 that is trimethylated HMT (Chaturvedi et al., 2012). Initially described as encoding a protein similar to G9a, G9a-like protein (GLP) has the same substrate specificity on histones as G9a. The G9a and GLP complex is associated with memory and learning, and participates in long-term potentiation, the maintenance of synaptic plasticity and upregulating Brain-derived neurotrophic factor (Sharma et al., 2017; Han et al., 2021). Treatment of transgenic mice with the G9a/GLP inhibitor UNC0642 reduces H3K9me2 and changes the overall levels of 5mC and 5hmC, increasing synaptic plasticity and reducing neuroinflammation, preventing Aβ plaque accumulation and enhancing cognitive function, thereby affecting the development of AD. Some research showed that inhibiting the activity of G9a/GLP may be a potential target for treating AD (Cao D. et al., 2020). Furthermore, the SET1 family of enzymes are well known for their involvement in H3K4 methylation. Thus, overexpression of the SET1/MLL family may be a potential cause of abnormally elevated H3K4me3 in the body. When the HMT function of SET1/MLL is inhibited, it can reduce the levels of H3K4me3 in the prefrontal cortex (PFC), restore the expression of glutamate receptor at synapses, increase PFC synaptic plasticity, and further improve cognitive impairment in AD mice. This suggests that targeting the SET1/MLL family of enzymes may also hold potential for the treatment of AD (Cao Q. et al., 2020). Proteins containing the Jumonji domain 3 (JMJD3) play a crucial role in activating neurogenesis in the adult brain ventricular zone by serving as histone H3K27 demethylases. High expression of JMJD3 leads to enhanced demethylation of H3K27me3 in related genes, such as DLX2, MLL1, and MASH1, thereby promoting neuronal differentiation (Cao D. et al., 2020). Previous studies showed inhibition of Lysine-specific histone demethylase 1 (LSD1) seems to be correlated with the activation of pluripotent genes and the induction of AD lesions. Moreover, research indicates that overexpression of LSD1 in hippocampal neurons can rescue cell death and inhibit the occurrence of inflammation (Engstrom et al., 2020).

### 4.3 ncRNAs

ncRNAs are closely related to the epigenetic mechanisms of AD and are diverse. In this article, we focus on miRNAs and lncRNAs (He et al., 2023). So far, many different miRNAs have been widely studied and linked with the development of AD, and the end result of the action of miRNAs has been the translational inhibition and/or degradation of targeted miRNAs (Huntzinger and Izaurralde, 2011). Some of miRNAs can lead to synaptic and cognitive dysfunction, and others can suppress neuronal apoptosis and pathological damage and improve the intelligence of AD mice (Gao et al., 2022). Several studies have found that MAPK/ERK activation is associated with the formation of miR-125 bp-tau, and the expression of miR-146 is closely related to NF-κB. Increased levels of miR-125 and miR-146 *in vivo* lead to tau protein aggregation. While miR-9 is involved in regulating calcium/calmodulin-dependent protein kinase 2 (CAMKK2), a decrease in its content can lead to Aβ plaque deposition (Chang et al., 2014). Moreover, some studies have shown that certain miRNAs are potential in AD therapy, it is still a new direction in AD research.

lncRNAs refer to a type of endogenous non-coding RNA with a length exceeding 200 nucleotides (Li and Wang, 2023). These lncRNAs are expressed in brain tissue and are distributed in the cell nucleus, cytoplasm, and mitochondria (Bridges et al., 2021). One of the most well-studied lncRNAs is BACE1-AS, which is transcribed from the reverse strand of the BACE1 gene locus on chromosome 11. It has been shown that upregulation of BACE1-AS may promote the generation of Aβ. Moreover, in the brains of AD patients, sustained production of Aβ can induce upregulation of BACE1-AS, thereby creating a positive feedback mechanism that further promotes Aβ expression and worsening the pathological deterioration (Li et al., 2019). Furthermore, in mouse experiments, down-regulation of BACE1-AS has been shown to improve memory in mice (Zhang et al., 2018). In addition to its role in AD development, BACE1-AS is also of interest in the autophagy-lysosomal system, where it promotes neuronal damage mediated by autophagy (Zhou et al., 2021). Another lncRNA of interest is brain cytoplasmic 200 (BC200), which is mainly expressed in the cell bodies and dendrites of neurons in the hippocampus and neocortex (Ding et al., 2022). BC200 can inhibit the expression of BACE1 and increase cell survival rates in AD cell models. Overexpression of BC200 in brain tissue elevates BACE1 expression, accelerating Aβ deposition (Li et al., 2018).

### 4.4 Prospects and limitations

Scientists believe that many natural compounds or their metabolites may have effects similar to those of known epigenetic regulators that can improve neurodegenerative diseases such as AD. For example, genistein, epigallocatechin gallate have the ability to inhibit DNMT and HDAC; lycopene and curcumin have antioxidant properties that delay aging (Castillo-Ordoñez et al., 2024). Moreover, postmenopausal women account for a higher proportion of women with AD (Barron and Pike, 2012). Loss of hormonal regulatory, synaptic, and neuroprotective effects likely contribute to AD pathology, and epigenetic regulation of hormones improved memory (Mills et al., 2023). This suggests that the epigenetic process of AD is closely related to estrogen, and estrogen receptors are widely distributed in nerve cells involved in learning and memory (Kumar et al., 2023).

The pathogenesis of most AD cases is generally considered to be a complex disease caused by a combination of multiple genetic and environmental factors, such as lifestyle, dietary habits, age, gender, education level, ethnicity, etc. The combination of these factors and their interaction may affect an individual’s sensitivity to AD risk. Therefore, through a comprehensive study of genetic and environmental factors, as well as an in-depth analysis of individual cases, a more comprehensive understanding of the pathogenesis of AD can provide more effective strategies for the prevention and treatment of AD (Sharma et al., 2020).

In the study of AD epigenetics, the correlation between DNA modification and histone modification and the combined effects of ncRNAs is not clear. Recent studies have shown that the wide application of omics technology in many fields such as epigenetics and genomics can collect many complex pathological data, and use its powerful computing and analysis capabilities to help discover more new biomarkers, which can eventually be used for AD research and related prediction and treatment (Pierouli et al., 2023; Tan et al., 2023). At present, it seems that the study of multiple omics data such as epigenomics, methylomics, transcriptomics will show an important role in determining the mechanism of epigenetics in AD in the future (Wang et al., 2023).

In recent years, the number of AD patients worldwide has been increasing, and with the development of new therapies, elucidating epigenetic mechanisms such as histone modification, DNA modification, and ncRNAs is crucial for the future development of safe and effective AD therapies (Zhang et al., 2022). The current clinical drugs for the treatment of AD are mainly pathological drugs targeting Aβ and tau proteins. This may be due to the lack of clinical trials of strategies such as neuroprotection, nerve cell suppression, immunomodulation, and protein homeostasis, and the high failure rate of drug clinical trials. In addition, many AD treatments are almost stuck in the experimental stage of animal models, and compounds recognized as disease-modifying pathways fail to enter clinical research without reason, thus hindering the development of clinical research (Dhapola et al., 2023; Jeremic et al., 2023). This requires re-examining the treatment of AD from various aspects. For example, first, to find new therapeutic targets. Second, to use multi-target therapy based on existing targets. In addition to classical Aβ and tau targets, a variety of targets such as cholinergic, glutaminergic, dopaminergic, GABAergic, adrenergic, and serotonergic signaling have been extensively studied as possible pathways for the treatment of AD (Kandimalla and Reddy, 2017). This trend of multi-target therapy provides new directions and possibilities for AD drug development, and it is hoped that more breakthroughs can be made in the future to bring more effective treatment options for AD patients. Third, to improve the current experimental technology, a variety of experimental methods such as multi-omics analysis, nanotechnology, etc. can be used for experiments (Dong et al., 2021; Cáceres et al., 2023). Generally speaking, in drug development related to epigenetics, advances in multi-target therapy should be the main focus of treatment for neurodegenerative diseases in the future (Ghosh and Saadat, 2023).

While there are many studies on the relationship between AD and epigenetics, there are still many challenges. Firstly, the epigenetic mechanism of AD has not been fully elucidated (Paniri et al., 2023). Although many studies have shown that AD is closely related to DNA methylation and hydroxy methylation, they have not really clarified the 5mC and 5hmC levels is the imbalance part of the cause of AD or just a consequence of AD (Nikolac Perkovic et al., 2021). Secondly, in the past 10 years, the number of AD patients has been increasing worldwide, but the treatment of AD has almost stayed in the experimental stage of animal models, and the trials in clinical application are insufficient. In the development of new drugs, the more detailed clarification of epigenetic mechanisms such as histone modification, DNA modification, and ncRNAs is crucial for the future development of safe and effective drugs for AD. Meanwhile, the current DNA methylation age estimation model lacks precision. The identification of a novel epigenetic clock capable of encompassing the entirety of human lifespan is a pressing concern within the field of DNA methylation research (Shireby et al., 2020). Additionally, it is important to acknowledge that epigenetic biomarkers, including miRNAs, do not comprehensively or accurately depict the pathological progression of AD (Ritchie et al., 2017). The primary challenge in utilizing miRNAs for external diagnosis lies in identifying specific miRNAs that can serve as markers for a diverse range of patients, as well as developing accurate, straightforward, and cost-effective methods to match them (Condrat et al., 2020).

This is the first bibliometric analysis of the epigenetics of AD, which has certain limitations. Firstly, the literature only sourced from the WOSCC, so the results obtained by the analysis are limited. Secondly, we have certain restrictions for screening the data. Recently published data and data other than papers and review papers published in English have not been analyzed. Finally, bibliometric analysis itself has certain limitations. It mainly quantitatively analyzes the characteristics of the literature and cannot analyze the details of the literature well, so the conclusions obtained are relatively superficial.

## 5 Conclusion

In order to explore the global development trend in the field of epigenetics of AD, we used bibliometrics to conclude that China is the most prolific country, Capital Medical University is the most prolific institution, Bennett is the most prolific and cited author. The current research focus is still to elucidate the epigenetic mechanism of AD in more detail from DNA modification, histone modification, ncRNAs, etc. and to find more specific biomarkers for the diagnosis of AD based on the above mechanisms. In terms of treatment, the comprehensive use of omics analysis technology to explore new therapeutic targets, and the development of drugs targeting HATs, TETs, HDACI, and RNA-based mechanisms for multi-target therapy should be the directions of future research.

## Author contributions

YZ: Visualization, Writing – original draft. WA: Visualization, Writing – original draft. JZ: Visualization, Writing – original draft. XH: Funding acquisition, Supervision, Writing – review & editing. LZ: Funding acquisition, Supervision, Writing – review & editing.
