# Supplementary material for: A bibliometric and visual analysis of epigenetic research publications for Alzheimer’s disease (2013–2023)
Source: Front Aging Neurosci. 2024 Jan 16;16:1332845. doi: 10.3389/fnagi.2024.1332845 (PMC10824959; doi:10.3389/fnagi.2024.1332845)
Supplement: Supplementary file 1 [file Table_1.DOCX]

**Screening strategies**

(((((((((((((((((((((((((((((((((((TS=(Alzheimer Disease )) OR TS=(Alzheimer Dementia)) OR TS=(Alzheimer Dementias)) OR TS=(Dementia, Alzheimer)) OR TS=(Alzheimer's Disease)) OR TS=(Dementia, Senile)) OR TS=(Senile Dementia)) OR TS=(Dementia, Alzheimer Type)) OR TS=(Alzheimer Type Dementia)) OR TS=(Alzheimer-Type Dementia (ATD))) OR TS=(Alzheimer Type Dementia (ATD))) OR TS=(Dementia, Alzheimer-Type (ATD))) OR TS=(Alzheimer Type Senile Dementia)) OR TS=(Primary Senile Degenerative Dementia)) OR TS=(Dementia, Primary Senile Degenerative)) OR TS=(Alzheimer Sclerosis)) OR TS=(Sclerosis, Alzheimer)) OR TS=(Alzheimer Syndrome)) OR TS=(Alzheimer's Diseases)) OR TS=(Alzheimer Diseases)) OR TS=(Alzheimers Diseases)) OR TS=(Senile Dementia, Alzheimer Type)) OR TS=(Acute Confusional Senile Dementia)) OR TS=(Senile Dementia, Acute Confusional)) OR TS=(Dementia, Presenile)) OR TS=(Presenile Dementia)) OR TS=(Alzheimer Disease, Late Onset)) OR TS=(Late Onset Alzheimer Disease)) OR TS=(Alzheimer's Disease, Focal Onset)) OR TS=(Focal Onset Alzheimer's Disease)) OR TS=(Familial Alzheimer Disease (FAD))) OR TS=(Alzheimer Disease, Familial (FAD))) OR TS=(Familial Alzheimer Diseases (FAD))) OR TS=(Alzheimer Disease, Early Onset)) OR TS=(Early Onset Alzheimer Disease)) OR TS=(Presenile Alzheimer Dementia) and TS = (epigenetic* OR epigenomic* OR DNA methylation" OR methyltransferase* OR demethylase* OR Hypermethylation OR Hypomethylation OR CpGisland" OR histone modification" OR histone methylation" OR histone phosphorylation" OR "histone deacetylase*" OR histone acetyltransferase*" OR chromatin remodeling" OR non-coding RNA*" OR MicroRNA* OR "micro RNAs" OR "micro RNA" OR micro- RNAs OR micro- RNA OR lncRNA* OR longncRNA*" OR "long noncoding RNA*" OR "Long Non-Coding RNA*" OR circRNA* OR Circular RNA*" OR "circ- RNA" OR "RNA modification" OR N6-methyladenosine OR 5-methylcytosine)
