# Supplementary figures and images for: A bibliometric and visual analysis of epigenetic research publications for Alzheimer’s disease (2013–2023)
Source: Front Aging Neurosci. 2024 Jan 16;16:1332845. doi: 10.3389/fnagi.2024.1332845 (PMC10824959; doi:10.3389/fnagi.2024.1332845)

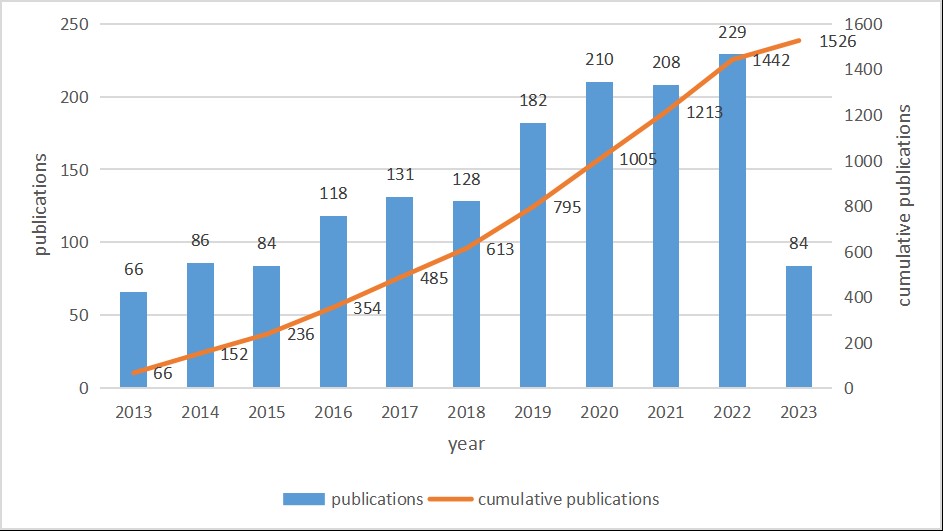

Supplement: Supplementary file 2 [file Image_1.JPEG]

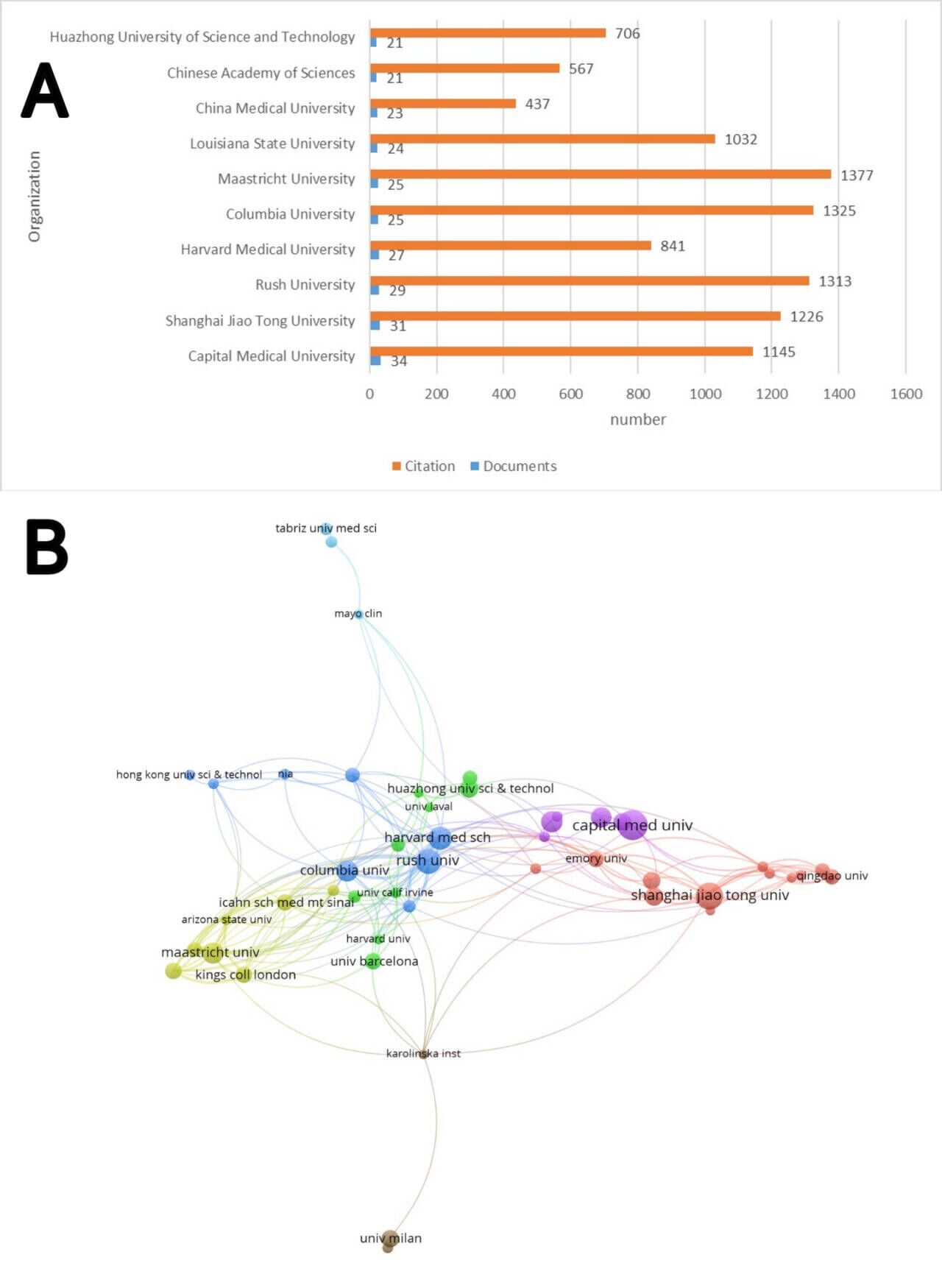

Supplement: Supplementary file 3 [file Image_2.JPEG]
